# Expression of FFAR3 and FFAR4 Is Increased in Gastroesophageal Reflux Disease

**DOI:** 10.3390/jcm9124111

**Published:** 2020-12-20

**Authors:** Adam Fabisiak, Adrian Bartoszek, Marcin Talar, Agata Binienda, Katarzyna Dziedziczak, Julia B. Krajewska, Paula Mosińska, Karolina Niewinna, Aleksandra Tarasiuk, Anna Mokrowiecka, Agnieszka Wierzchniewska-Ławska, Ewa Małecka-Panas, Maciej Salaga, Jakub Fichna

**Affiliations:** 1 Department of Biochemistry, Faculty of Medicine, Medical University of Lodz, 92-215 Lodz, Poland; adam.fabisiak@umed.lodz.pl (A.F.); adrianbartoszek96@gmail.com (A.B.); marcin.talar@umed.lodz.pl (M.T.); agata.binienda@gmail.com (A.B.); katarzyna.dziedziczak@umed.lodz.pl (K.D.); krajewska.julia@gmail.com (J.B.K.); paula.mosinska@gmail.com (P.M.); karolina.niewinna@umed.lodz.pl (K.N.); tarasiuk.aleksandra@gmail.com (A.T.); maciej.salaga@umed.lodz.pl (M.S.); 2 Department of Digestive Tract Diseases, Faculty of Medicine, Medical University of Lodz, 90-153 Lodz, Poland; anna.mokrowiecka@umed.lodz.pl (A.M.); ewa.malecka-panas@umed.lodz.pl (E.M.-P.); 3 Department of Pathology, Faculty of Medicine, Medical University of Lodz, 92-213 Lodz, Poland; agnieszka.wierzchniewska-lawska@umed.lodz.pl

**Keywords:** free fatty acid receptor, gastroesophageal reflux disease, inflammation

## Abstract

Background: The negative impact of a high-fat diet on the course of gastroesophageal reflux disease (GERD) has been previously reported. Free fatty acid receptors (FFARs) may be mediators of this phenomenon. The aim of this study was to characterize the role of FFARs in the course of nonerosive (NERD) and erosive (ERD) reflux disease. Methods: Collectively, 73 patients (62 with GERD and 11 healthy controls (HCs)) were recruited to the study. Esophageal biopsies were drawn from the lower third of the esophagus and kept for further experiments. Quantitative, real-time polymerase chain reaction was used to assess the expression of FFAR1, FFAR2, FFAR3, and FFAR4 in biopsies. Histological evaluation of dilated intracellular spaces (DISs) was also performed. Results: FFAR3 exhibited the highest expression, and FFAR4 exhibited the lowest expression in all esophageal samples. Higher relative expression of FFAR1 and FFAR2 and significantly higher expression of FFAR3 (*p* = 0.04) was noted in patients with GERD compared to respective HCs. Patients with nonerosive GERD (NERD) presented higher expression of all FFARs compared to patients with erosive GERD (ERD) and respective HCs. Interestingly, in patients with ERD, the expression of FFAR3 was lower than in HCs. Significant, weak, positive correlation was found for FFAR3 and FFAR4 expression and DIS scores (r = 0.36, *p* < 0.05 for FFAR 3, and r = 0.39, *p* < 0.05 for FFAR4). Conclusions: In this study, we show that FFARs may play a role in GERD pathogenesis, particularly in the NERD type. It may be assumed that FFARs, in particular FFAR3 and FFAR4, may have diagnostic and therapeutic potential in GERD.

## 1. Introduction

Gastroesophageal reflux disease (GERD), also known as acid reflux, is a chronic disorder of the digestive tract characterized by a wide range of symptoms depending on the severity of the condition. In accordance with the Montreal definition, GERD is a state that develops when the reflux of stomach contents causes troublesome symptoms and/or complications [1].

The incidence of GERD is estimated to be the highest in North America (18.1–27.8%), Europe (8.8–25.9%), and the Middle East (8.7–33.1%), followed by South America (23%), Australia (11.6%), and East Asia (2.5–7.8%), which makes it one of the most common chronic diseases [1]. Many studies pointed out a growing number of GERD cases in Asia [2,3]. It was shown [4] that GERD severely impairs patients’ quality of life.

The pathophysiology of the disease, above all, includes low resting pressure of the lower esophageal sphincter (LES). Various factors lead to this pathology, such as hypotension and transient relaxations of LES, anatomic disruptions of the gastroesophageal junction, delayed gastric emptying, and others [5]. Diets high in salted or fatty foods [6] and obstructive sleep apnea [7] have been associated with changes in LES pressure. 

The diagnosis of GERD is predominantly symptom-based, and is confirmed by a positive response to acid-suppression therapy. The monitoring of esophageal pH as well as endoscopic and histological examinations complement the proton pump inhibitor (PPI) test in cases where diagnosis is uncertain [5]. Up to 70% of patients with GERD present no mucosal changes in the esophagus; this lack of change is related to nonerosive reflux disease (NERD), while patients with erosive reflux disease (ERD) develop macroscopic lesions in the lower third of the esophagus. The pathogenesis of the former is much less well-understood, but several factors including visceral hypersensitivity and neurogenic inflammation have been suggested to be implicated in NERD [8]. In addition, the diagnosis of NERD is much more complex, requiring further tests to rule out functional diseases of the esophagus. An important feature of both subtypes is the presence of dilated intracellular spaces (DISs), observed while assessing the histopathological samples from patients with GERD [9]. DISs are the distinctive sign of mucosal injury from reflux [10]. 

Long-term acidic esophageal injury in the course of GERD may lead to a precancerous state— Barrett’s esophagus (BE) and/or further gastroesophageal junction adenomatous cancer [11,12].

Unfortunately, treatment of the disease is still only symptomatic, with PPIs, lifestyle modifications, and laparoscopic fundoplication being proven methods [13]. 

A high-fat diet—saturated fat in particular—can cause frequent reflux episodes as well as an increased risk of GERD symptoms [14]. In a large-scale case–control study, a relationship between high fat intake and both ERD and NERD was found [15]. On the other hand, an epidemiological, large-population study showed no significant relation between fat-rich diets and GERD risk [16]. Therefore, the role of high fat intake in GERD pathogenesis and the clinical course is not yet fully elucidated.

We hypothesize that receptors which bind free fatty acids (FFAs) may play a role in GERD’s development and clinical course. The family comprises four receptors: FFAR1, FFAR2, FFAR3, and FFAR4 [17]. FFAs are important modulators of many cellular functions, such as proliferation, migration, and apoptosis, as well as the production of cytokines and hormones [18]. FFARs are present in many tissues, including intestinal enteroendocrine cells [19], central nervous system cells [20], and immune cells [21]; however, their presence in the upper part of the human gastrointestinal (GI) tract has not been explored. 

The aim of this study was to investigate the expression of FFARs in esophageal samples of patients suffering from GERD and healthy controls (HCs), and to investigate the possible relationship between FFAR expression, endoscopic changes, and the histological assessment of dilated intracellular spaces (DISs).

## 2. Methods

### 2.1. Study Population

Patients recruited to the study were admitted for upper GI endoscopy for different indications in the Department of Digestive Tract Diseases at the Barlicki Memorial Hospital in Lodz, Poland in 2019 (approval of bioethical committee no. RNN/12/19/KE). Inclusion criteria encompassed patient history of GERD, diagnosed based on the positive response to a PPI test. Exclusion criteria were as follows: (i) any other inflammatory disease of the gastrointestinal (GI) tract, (ii) Barrett’s esophagus, and (iii) gastric or esophageal neoplasia. Patients were given the consent form for participation in the study and questionnaires to fill out. Furthermore, biopsies from the lower third of the esophageal mucosa were taken during the procedure for biochemical and histological assessment. The samples were kept on ice and transferred to the Department of Biochemistry, Medical University of Lodz, Poland where they were stored at −80 °C for further analyses. Collectively, we managed to recruit 73 patients: 62 with GERD and 11 HCs. Four patients from the GERD group were excluded, and their biopsies and documents were destroyed after the initial review due to the recognition of endoscopically suspected esophageal metaplasia. Los Angeles classification was used to measure the grade of esophagitis. Among GERD patients, 41 patients had nonerosive reflux disease (NERD), and 17 patients had erosive reflux disease (ERD). Sixteen ERD patients were classified as grade A, and one patient was classified as grade B; no instances of grade C or D were observed. 

### 2.2. Expression of FFARs in Esophageal Mucosa

- RNA isolation: Samples were isolated according to the manufacturer’s protocol using a Total RNA Mini kit (A&A Biotechnology, Gdynia, Poland). Briefly, tissue samples were homogenized in TRIsure reagent (Bioline, UK) using an ultrasound homogenizer (Bandelin Sonoplus HD3100, Germany). The purity and quantity of the isolated RNA were measured using a Colibri Microvolume Spectrometer (Titertek Berthold, Colibri, Germany). Total RNA was eluted using diethyl-pyrocarbonate-treated water.
- Reverse transcription: cDNA synthesis was performed with the RevertAid First Strand cDNA Synthesis Kit (Fermentas, Canada) in accordance with the manufacturer’s protocol. Total RNA (1 µg) was used in the reverse transcription reaction in a total volume of 20 µL with the following four-step incubation: 25 °C for 10 min, 50 °C for 15 min, 85 °C for 5 min, and 4 °C for 10 min.
- Quantitative real-time RT-PCR: For the quantification of mRNA expression, we applied the real-time fluorescence detection PCR method with FAM dye-labeled TaqMan probes: FFAR1 (Hs03045166_s1), FFAR2 (Hs00271142_s1), FFAR3 (Hs02519193_g1), FFAR4 (Hs00699184_m1) (Thermofisher, Waltham, USA). Values obtained for studied genes were normalized to the expression of the hypoxanthine phosphoribosyltransferase 1 (HPRT1) gene (Hs02800695_m1, Thermofisher, Waltham, USA) as an endogenous control. The real-time reaction mixture was prepared in a total volume of 10 µL and consisted of 0.5 µL cDNA, 5 µL TaqMan Gene Expression Master Mix, 0.5 µL TaqMan Gene Expression Assays, and 4 µL RNA-free water; this was performed in triplicate. The cDNA was amplified in a LightCycler (Roche, Switzerland). Cycle parameters were as follows: initial denaturation at 95 °C for 10 min, followed by 40 cycles of sequential incubations at 95 °C for 15 s and at 60 °C for 1 min. The initial amount of the template was evaluated as a Ct parameter. The number of cycles linearly correlates with the logarithmic value of RNA quantity. The relative expression level normalized to HPRT1 was calculated as 2 − (CtFFARx − CtHPRT1) × 1000.

### 2.3. Histological Assessment of Dilated Intracellular Spaces (DISs)

The DIS score was evaluated during the routine microscopic assessment of esophageal samples. Briefly, after the collection, specimens were fixed in 10% neutral-buffered formalin for 24 h at 4 °C. After subsequent dehydration in sucrose, the samples were embedded in paraffin, sectioned at 5 μm, and mounted onto slides. Then, sections were stained with hematoxylin and eosin and examined using an Olympus CX43. The severity of DISs was scored in one high-power field as follows: 0 (absent; ≤5 small intercellular spaces), 1 (≥6 small intercellular spaces and ≤5 large intercellular spaces), or 2 (≥6 large intercellular spaces), where small was defined as narrower than one lymphocyte in diameter and large was as equal to or wider than one lymphocyte in diameter. DISs near the periphery of a biopsy may be artifactual, and were disregarded in this evaluation.

### 2.4. Statistical Analyses

Statistical analysis was performed using Prism 8.0 (GraphPad Software Inc., La Jolla, CA, USA). Assumption of the normal distribution of differences was verified with the use of the Shapiro–Wilk test. As the normality assumption was violated, the significance of differences was tested with Mann–Whitney’s U test to compare two independent groups. For multiple comparison, the Kruskal–Wallis test was applied. The data are expressed as median with interquartile range. Analysis of the correlation between FFARs expression and DIS score was conducted by calculation of Spearman’s rank correlation coefficient. A heatmap showing the relation between the relative expression of FFARs 1–4 and DISs score was presented in mean values. Outliers were counted using the ROUT method and excluded. *p*-Values < 0.05 were considered statistically significant. 

## 3. Results

### 3.1. The mRNA Expression of FFAR1, FFAR2, and FFAR3 was Increased in Patients with GERD Compared to HCs

**Figure 1:**
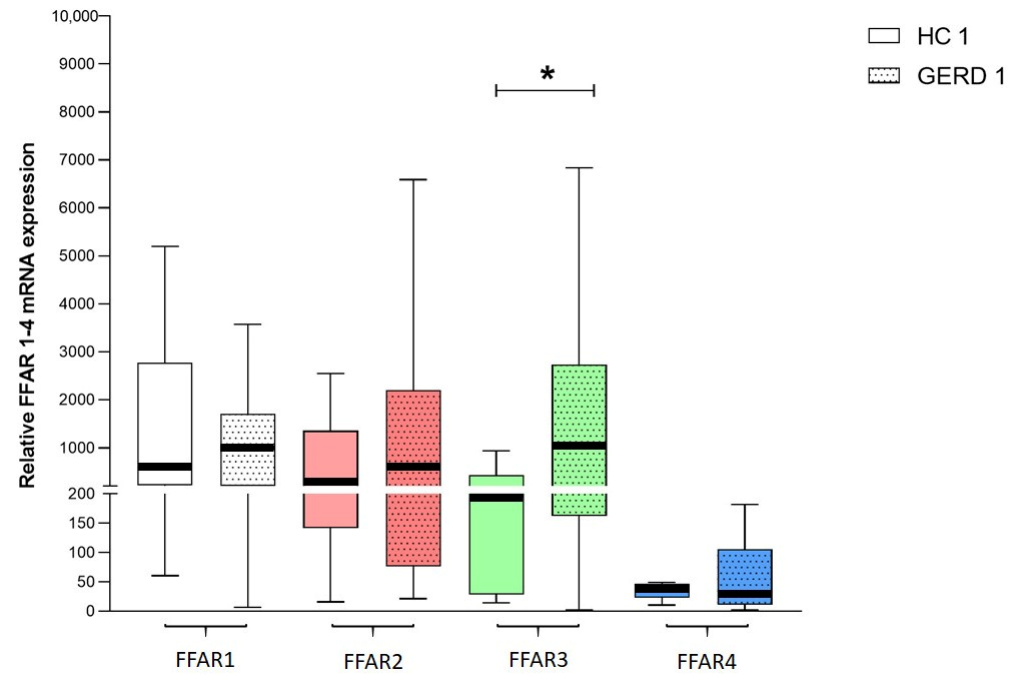
The relative expression of FFAR1, FFAR2, FFAR3, and FFAR4 in healthy controls (HCs) (HC1–4, *n* = 8) and patients with gastroesophageal reflux disease (GERD) (GERD 1–4, *n* = 47–51). Expression was compared with the HPRT1 reference gene. The Mann–Whitney test was used to compare the values. * *p* < 0.05 as compared to respective control.

The median relative expression of FFAR1, FFAR2, and FFAR3 was higher in esophageal biopsies obtained from patients with GERD than in respective HCs; however, statistical significance was reached only for FFAR3 (979 (203–1705) vs. 543 (210–2780) for FFAR1, 552 (76–2205) vs. 337 (141–1361) for FFAR2, 1030 (161–2732) vs. 197 (28–435) for FFAR3 (*p* < 0.05)) (Figure 1). Conversely, FFAR4 was less abundant in patients with GERD than in HCs (30 (10–105) vs. 39 (22–47)). FFAR3 presented the highest expression, whereas FFAR4 presented the lowest expression in patients with GERD.

### 3.2. Patients with NERD Exhibited Higher mRNA Expression of FFARs Compared with HCs and Patients with ERD

**Figure 2:**
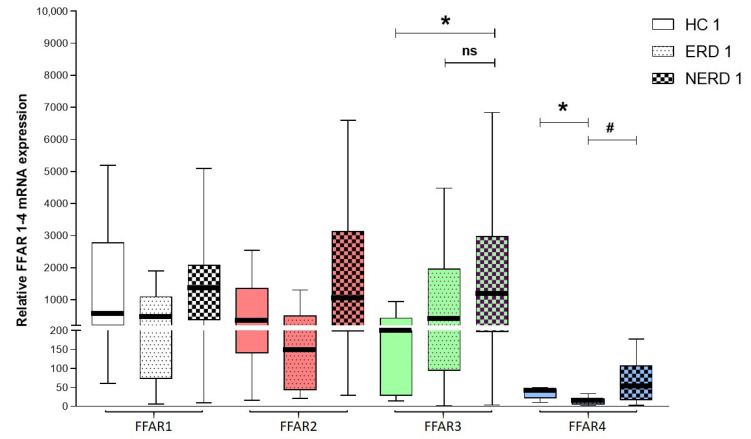
The relative expression of FFAR1, FFAR2, FFAR3, and FFAR4 in HCs (HC1–4, *n* = 8), patients with erosive reflux disease (ERD) (ERD 1–4, *n* = 12-15), and patients with nonerosive reflux disease (NERD) (NERD 1–4, *n* = 32-36). Expression was compared with the HPRT1 reference gene. The Kruskal–Wallis test was used to compare the values. ns—non significant * *p* < 0.05 as compared to respective control, # *p* < 0.05 as compared to ERD.

The median expression of FFAR1, FFAR2, FFAR3, and FFAR4 was higher in NERD than in ERD patients and respective HCs (1357 (378–2081) vs. 442 (73–1097) vs. 543 (210–2780) for FFAR1, 1035 (198–3135) vs. 147 (43–504) vs. 337 (141–1361) for FFAR2, 1183 (197–2983) vs. 416 (94–1966) vs. 197 (28–435) for FFAR3 (*p* < 0.05 when comparing the expression in NERD to HCs), and 51 (17–107) vs. 12 (6–20) vs. 39 (22–47) for FFAR4 (*p* < 0.05 when comparing the expression in ERD to NERD and HCs)). The median expression of FFAR3 was higher in ERD patients than in HCs (Figure 2).

### 3.3. Patients with GERD Presented a Specific DIS Pattern

**Figure 3:**
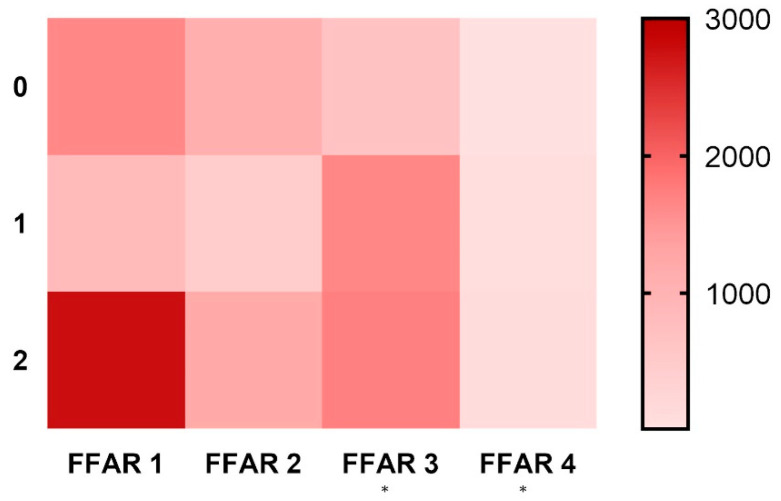
Heatmap showing the relation between the relative expression of FFARs 1–4 (*x* axis) and DIS score (*y* axis) (*n* = 24–30). Heat legend is located on the right side of graph. Calculated using the Spearman rank correlation test, * *p* < 0.05.

When the mean expressions of FFARs and DISs scores in esophageal samples were compared, we found significant, weak, positive correlations for FFAR3 (648 for DIS 0, 1647 for DIS 1, and 1710 for DIS 2; r = 0.36, *p* < 0.05) and FFAR4 (12, 68, and 98, respectively; r = 0.39, *p* < 0.05). The expression of FFAR1 (1636 for DIS 0, 834 for DIS 1, and 2759 for DIS 2) and FFAR2 (1087, 428, and 1178, respectively) was lower in the DIS 1 group than in the DIS 0 and DIS 2 groups (Figure 3). The expression of FFAR1 in patients characterized by DIS 2 was significantly higher than in DIS 1.

## 4. Discussion

Free fatty acids exert multiple functions throughout the human body, affecting cell migration, proliferation, apoptosis, and the production of reactive oxygen species, nitric oxide, eicosanoids, cytokines, and hormones. There are also reliable reports on the anti-inflammatory properties of free fatty acids in asthma [18], inflammatory bowel diseases [22], and cancer, including colorectal cancer [23]. There is a lack of studies regarding the potential role of free fatty acids and their receptors in GERD. In our study, we shed light upon the potential role of FFARs in this disorder.

In this study, we observed the overall trend of increased expression of FFAR1, FFAR2, and FFAR3 in GERD patients compared with HCs. The most noticeable differences were noted for FFAR3, and it was further revealed that the NERD subtype was the most accountable for this phenomenon. Interestingly, no correlation was observed between the FFAR expression and patients’ perception of the symptoms, regardless of the disease subtype. The results we obtained correspond with the study of Tsai et al. [24], showing that the selective agonists of FFAR1 (fasiglifam, TUG424) induce the relaxation of porcine LES strips ex vivo in a dose-dependent manner.

Interestingly, similarly to Tsai et al. [24], we observed a low expression of FFAR4 in esophageal samples. In line with our observations, they found that FFAR4 agonists (GW9508 and GSK137647) were less potent in relaxing the LES than those of FFAR1. 

We also observed that patients with ERD presented significantly lower expression of FFAR4 than subjects with NERD and HCs. A corresponding trend was found in in vitro studies performed by Muredda et al. [25] where, in adipocytes incubated in an inflammatory milieu containing interleukin 1β (IL-1β) and tumor necrosis factor α (TNF-α), the expression of FFAR3 was enhanced and FFAR4 expression was suppressed. This relation was achieved in our experiment where FFAR3 was more abundant and FFAR4 was less abundant in esophageal biopsies of patients with ERD than in respective controls. Importantly, Souza et al. [26] proposed a mechanism in which the refluxate causes cytokine-mediated inflammatory injury associated with increased nuclear factor kappa B (NF-kB)/p65 activity and the augmented expression of IL-8, IL-1β, TNF-α, cyclooxygenase-2, and intercellular adhesion molecule-1. Accordingly, the activation of FFAR4 suppresses the nuclear translocation of NF-kB 27, reduces IL-1β levels by enhancing cellular autophagy [27], and downregulates TNF-α signaling [28]. A clear connection between the immunological features of GERD and the mechanisms of FFAR4-mediated pathways makes the receptor a potential target in the development of a future therapeutic approach. Moreover, as FFAR4 was found to be significantly elevated in human esophageal cancer cells [29] serving as a positive regulator of malignant transformation, it may appear as an attractive early biomarker of esophageal carcinogenesis—especially in patients with ERD and BE. However, the studies regarding the utility of FFARs in BE are still lacking.

The discrepancies in the results between ERD and NERD subpopulations evidenced in our study may be due to the heterogeneity seen in the latter group, and reflect the pathogenetic differences observed between these two subgroups [30]. Entities such as acid- and non-acid-hypersensitive esophagus may be concealed among patients diagnosed with NERD. Although our NERD subpopulation consisted entirely of PPI responders, it has to be noted that 4% of patients with functional heartburn (FH) also respond positively to the PPI test as shown in a study by Savarino et al. [31]. That said, our study group could be biased with patients with FH, as the 24 h impedance-pH testing was not conducted in every patient. The prevalence of DISs in patients with FH is rather low and comparable to HCs when opposed to NERD [32]. Based on the assumption that patients who achieved DIS 0 in this study can be regarded as FH-predominant, we can assume that they would present a distinctive pattern of FFARs expression (FFAR1 > FFAR2 > FFAR3 > FFAR4), implying another pathophysiological difference from other subtypes of NERD. However, further studies directed precisely at specific subgroups of NERD are needed to explain this phenomenon.

In our study, histological assessment showed that the expression of FFAR3 and FFAR4 significantly correlated with the severity of microscopic damage in GERD assessed as DISs intensity in histopathological samples. Despite the very weak correlation we obtained, the evidence supporting our findings can be found in the literature. Generally, increased esophageal permeability in GERD is associated with E-cadherin cleavage [33], and it was known beforehand that short-chain FAs that are also involved in the signal transduction in FFAR2 and FFAR3 [34,35] up-regulate the transcription of E-cadherin [35]. In addition, the beneficial effect of monobutyrin (butyric acid derivative) in improving the intestinal permeability, as shown by Nguyen et al. [36] in rats, advocates for the use of FFAR3 as a potential therapeutic target in GERD.

To our knowledge, this is the first study considering the changes in FFARs expression in the esophagus of GERD patients. Our findings underline the importance of broadening the research on potential dietary indications in patients with GERD. Our study suffers the limitation of a rather vast disproportion between the study groups (GERD > HCs), which can be considered a significant drawback. Even though DISs are not considered the sole histological marker of GERD [37], the grading system allowed us to provide information that strengthened our observations.

## 5. Conclusions

Herein, we presented a potential role of FFARs in GERD. Two particular candidates, FFAR3 and FFAR4, appeared as plausible new targets that should be further evaluated in the field of GERD. Notably, our results suggest that FFARs may be particularly involved in the course of the NERD subgroup of patients. Collectively, with reports on visceral hypersensitivity, these outcomes fill the gap between the role of dietary fat content and the pathophysiology of NERD. We hope our work will act as a prelude to a deeper investigation into the role of FFARs not only in GERD, but also in other esophageal diseases.
